# Accessibility to molecular studies in gliomas: a challenge for precision medicine in low-resource countries

**DOI:** 10.3389/fonc.2026.1731276

**Published:** 2026-04-20

**Authors:** Pablo S. Paolinelli, Tomas Saavedra Azcona, Florencia B. Casto, Ezequiel Jungberg, Nicolás Tilano, William A. Blettler, Joan S. Pazos, Silvina Dell’Era, Clara Lynch, Miguel Villaescusa, Pedro Plou, Pablo Ajler

**Affiliations:** 1 Neurosurgical Department, Hospital Italiano de Buenos Aires, CABA, Buenos Aires, Argentina; 2 Research Department, Instituto Universitario Hospital Italiano de Buenos Aires, CABA, Buenos Aires, Argentina; 3 Faculty of Engineering, Universidad Austral, Pilar, Buenos Aires, Argentina

**Keywords:** glioma, Latin America, low resources, molecular diagnostics, molecular parameters, precision medicine, WHO classification

## Abstract

Background: CNS tumors present significant diagnostic challenges due to their heterogeneity. Successive editions of the World Health Organization (WHO) classification have progressively incorporated molecular markers as essential criteria. Nonetheless, in low- and middle-income countries, limited access to molecular testing hampers the full application of these classifications. To assess this situation, we decided to evaluate the impact of successive WHO classifications on glioma diagnostic accuracy in a high-volume neurosurgical center in Latin America. We conducted a retrospective analysis of patients that underwent elective tumor surgery between January 1st, 2010 and December 31st, 2024 and whose pathology report was consistent with a glioma. Demographic, anatomical, histological, and molecular data were collected. Tumors were classified according to WHO CNS Tumor Classification criteria of 2007, 2016, and 2021. Statistical analyses were performed using STATA v15.A total of 443 patients were included. Under WHO 2007 criteria, glioblastoma was the most common tumor type, with virtually all tumors classified histologically. With WHO 2016 implementation, the proportion of tumors labeled as “not otherwise specified” (NOS) and cases with incomplete tumor characterization increased. Application of WHO 2021 further highlighted diagnostic limitations, with 24% of tumors categorized as high- or low-grade glioma NOS, instead of a particular tumor type/entity. This work sheds light on the fact that the progressive complexity of glioma classification with the addition of new molecular and genetic factors may have as a counterpart an increase in the number of cases with inadequate tumor characterization due to the lack of accessibility to key molecular studies in diagnosis.

## Introduction

Central nervous system (CNS) tumors are a significant cause of morbidity and mortality across all age groups, with no clearly established risk factors to explain their occurrence (1). Epidemiologically, in the United States, the age-adjusted average annual incidence rate for all brain and other CNS tumors was 24.83 per 100,000 inhabitants between 2016 and 2020, with 6.94 per 100,000 for malignant tumors and 17.88 per 100,000 for non-malignant tumors (2). Among primary CNS tumors, gliomas are the most common histological type, accounting for approximately 80–85% of all brain tumors (3). Glioblastoma, the most frequent glioma subtype, is associated with markedly poor survival (4, 5).

Primary brain tumors are remarkably diverse, arising from virtually any cell type in the central nervous system. Gliomas are thought to arise from multipotent progenitor or stem-like cells within the central nervous system, although their exact cell of origin remains incompletely understood. Other types include meningiomas from the meninges, embryonal tumors like medulloblastomas, primary CNS lymphomas, germ cell tumors, and a range of nerve sheath and mesenchymal tumors. This wide variety reflects the complex cellular makeup of the brain and leads to significant differences in biological behavior, prognosis, and treatment strategies.

The WHO Classification of CNS Tumors is the standard by which CNS neoplasms are organized. Over the past twenty years, this system has changed significantly. The 2007 edition (and earlier ones) relied solely on histologic criteria (6). In 2016, important molecular markers were included for the first time (7). Finally, the latest version—the 2021 5th edition—went a step further, requiring molecular profiling for the accurate diagnosis of many tumor types (7). This shift represented a profound paradigm change, reflecting a far more precise understanding of tumor biology and pathogenesis. However, while this molecularly integrated classification improves diagnostic accuracy and guides personalized therapy, it also demands advanced technology, specialized resources, and expert interpretation—challenges that remain significant barriers in many regions of the world where such infrastructure is still limited or unavailable. This constraint, which reflects a common issue in developing countries, hampers the full implementation of the recent WHO classification criteria, which require precise molecular information to accurately categorize gliomas and other types of CNS tumors. Even in centers with high-level surgical and pathological resources, the lack of internal molecular platforms results in reliance on external laboratories, causing delays, increased costs, or even the unavailability of certain analyses (8). As a result, pathology, oncology, and neurosurgery professionals are constrained in their ability to assign definitive molecular diagnoses, which negatively impacts treatment decisions and prognostic stratification.

We hypothesized that the progressive incorporation of mandatory molecular markers in successive glioma CNS WHO classifications would be associated with an increased frequency of incomplete tumor characterization in a resource-limited setting due to inadequate access to molecular testing.

To that end, we decided to assess the challenges faced by a high-volume tertiary reference center in Latin America in achieving accurate glioma diagnosis. Therefore, we proposed to compare the proportion of accurate diagnoses achieved according to the criteria established in the WHO classifications of 2007, 2016, and 2021 in patients operated at a tertiary neurosurgical center during the period between 2010 and 2024.

## Materials and methods

A retrospective cross-sectional study was conducted in the department of neurosurgery of a high-complexity center with a large volume of patients with central nervous system tumors. All patients over 18 years of age with a histopathological and/or molecular diagnosis of glioma who underwent elective surgical intervention between 2010 and 2024 were included. Other primary CNS neoplasms different than those from astrocytic or oligodendrocytic lineage or those lacking sufficient information to determine tumor classification were excluded.

A consecutive sampling method was applied, including all patients who met the selection criteria during the study period. Given the retrospective nature of the study and the inclusion of the entire eligible population, no sample size calculation was performed.

The entire cohort was divided into 3 groups, according to the WHO’s Classification Criteria corresponding to each period. The cases of gliomas operated on between January 1, 2010, and December 31, 2016, correspond to the cohort classified according to the CNS WHO tumor classification 2007 (3rd edition) criteria. The cases operated on between January 1, 2017, and December 31, 2021, correspond to the cohort classified according to the CNS WHO tumor classification 2016 (4th edition) criteria. The cohort of cases operated on between January 1, 2021, and December 31, 2024, correspond to the cohort classified according to the CNS WHO tumor classification 2021 (5th edition) criteria. This division is in line with the fact that the complete implementation of the changes proposed by each update of the classification occurred the year following its publication, at the center where the study was performed.

The collected variables include patient characteristics (age and sex), tumor location, histological grade (1 to 4), and WHO classification of 2007, 2016 or 2021. Available immunohistochemical and molecular markers were also recorded, including: *IDH-1/2* (Isocitrate dehydrogenase 1/2), ATRX (alpha-thalassemia/mental retardation, X-linked), TP53, EGFR (Epidermal growth factor receptor) amplification, 1p/19q codeletion, BRAF V600E mutation and MGMT (O6-Methylguanine-DNA methyltransferase) activity. Data were obtained from the institutional electronic medical records, whose validity was ensured through double review and systematic quality control procedures.

All histopathological evaluations were performed at the institutional pathology laboratory, which holds accreditation from the College of American Pathologists (CAP). To minimize inter-observer variability, all cases included in this study were reviewed and diagnosed by the same senior neuropathologist.

Tumor samples were fixed in 10% buffered formalin and embedded in paraffin for immunohistochemical (IHC) studies, and these were performed using an automated staining platform (BenchMark XT Medical Systems) with the OptiView DAB IHC Detection Kit. The routine immunohistochemical panel included GFAP, OLIG2, IDH1 R132H, P53, ATRX, and Ki-67. Although these markers were generally available throughout the study period, their use in individual cases could vary depending on reagent availability.

IDH mutation status was assessed by immunohistochemistry for IDH1 R132H. Sequencing for non-canonical *IDH1* or *IDH2* mutations was performed only upon specific clinical or pathological indication. Prior to 2024, sequencing was performed using the Oncomine Focus Assay (Thermo Fisher Scientific), while from 2024 onward, the Idylla™ Biocartis system became available. *EGFR* amplification and 1p/19q codeletion were assessed by fluorescence *in situ* hybridization (FISH). These studies were not performed routinely and were requested selectively based on histopathological findings, diagnostic uncertainty, or potential therapeutic implications. BRAF V600E mutation analysis was performed upon request using the COBAS 4800 system (Roche). *TP53* status was inferred through P53 immunohistochemical expression patterns, according to standard neuropathological practice. Although *EGFR* and *TP53* mutational status can be assessed through sequencing, this was not performed routinely during the study period. *MGMT* activity was assessed exclusively by immunohistochemistry using the same automated platform described above when reagents were available. Assessment of *MGMT* promoter methylation by DNA-based methods was not locally available during the study period and was therefore not performed in any case.

Tumors were classified as “not otherwise specified” (NOS) or as having an indeterminate or incomplete integrated diagnosis when essential molecular information required by the corresponding WHO classification could not be obtained. In these cases, tumor lineage and histological grade were determined based on conventional morphology and available immunohistochemical markers; however, the absence of mandatory molecular alterations precluded assignment of a fully integrated WHO diagnosis.

For the purposes of this study, we distinguished between formal WHO diagnostic categories and a broader concept of diagnostic completeness. While the term “not otherwise specified” (NOS) has a specific definition within the WHO 2016 and 2021 classifications, our primary analytical focus was on gliomas lacking a fully integrated diagnosis due to missing mandatory molecular data. Accordingly, throughout the analyses we refer to these cases as “incompletely characterized” or “indeterminate” gliomas, a category that includes formal WHO-NOS diagnoses when applicable, as well as tumors in which required molecular information could not be obtained. This approach avoids retroactive application of modern WHO terminology to earlier periods while allowing longitudinal assessment of diagnostic feasibility across classification systems.

Statistical analysis was carried out using STATA software, version 15 (StataCorp LP, College Station, TX, USA).

The study protocol was approved by the Research Ethics Committee of our institution. As this was a retrospective study with minimal risk and no direct patient contact, a waiver of informed consent was requested and granted. All data were anonymized in accordance with the National Personal Data Protection Law (Law No. 25.326) and international confidentiality standards (HIPAA).

## Results

A total of 443 patients with a diagnosis of glioma who underwent surgery at our center during the study period were included. The sex distribution showed a slight male predominance, with 236 males (53.3%) and 207 females (46.7%). Patient age ranged from 18 to 87 years, with a median of 61 years (interquartile range: 49–71).Regarding anatomical location, most tumors were supratentorial (94.6%; n=419), while a smaller proportion were infratentorial (5.0%; n=23) or involved both supra- and infratentorial regions (0.2%; n=1).

**Figure 1:**
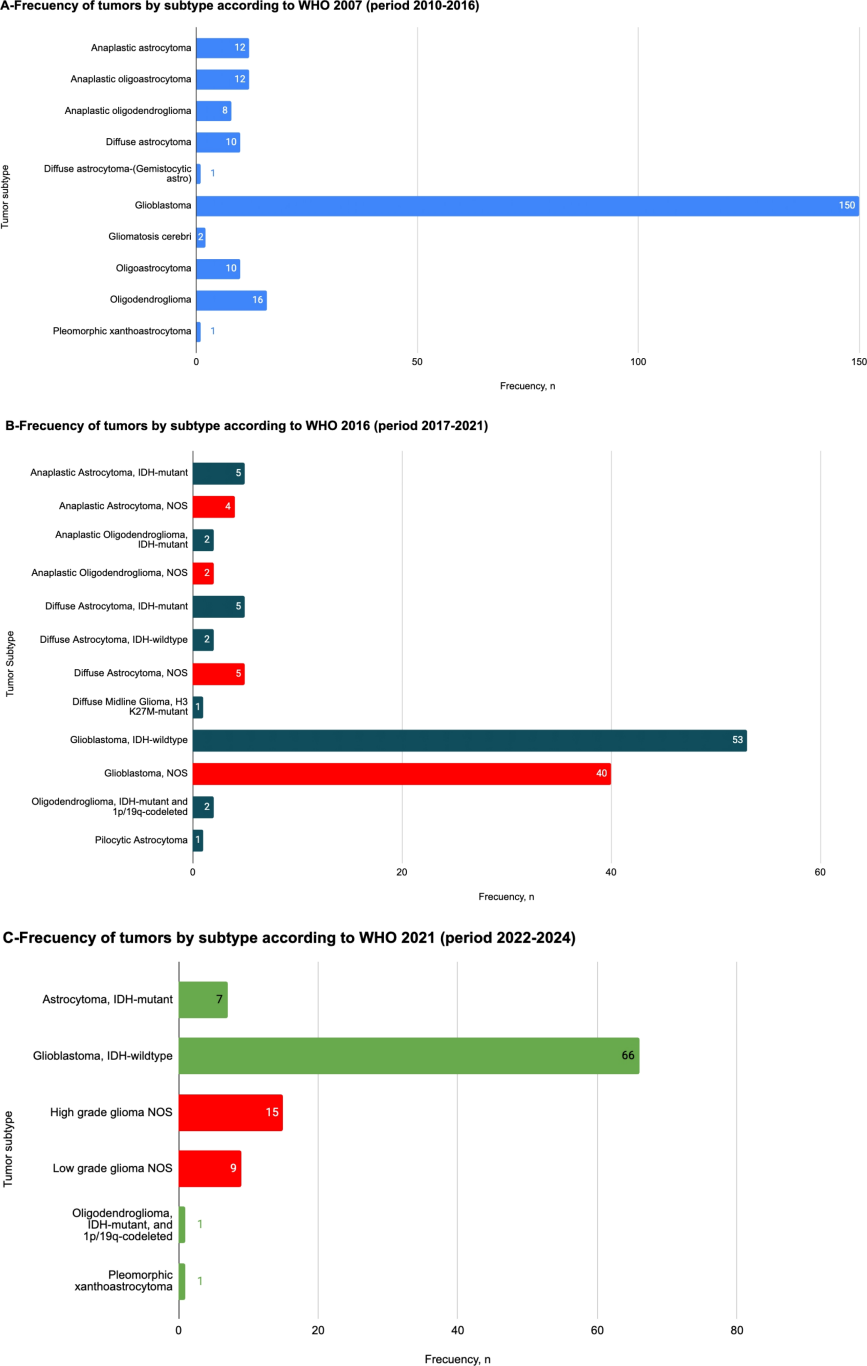
**(A)** Frequency of glioma subtypes according to the 2007 WHO classification in a cohort of 222 patients operated from 2010 to 2016. Glioblastoma was the most frequent tumor type (n=151), including 15 cases of giant cell glioblastoma and 4 cases of gliosarcoma. In decreasing order of frequency, remaining tumor types included Anaplastic astrocytoma (n=14), Anaplastic oligoastrocytoma (n=13), Oligodendroglioma (n=16), Diffuse astrocytoma (n=9), Gliomatosis cerebri (n=2), and Pleomorphic xanthoastrocytoma (n=1). No indeterminate diagnosis were reported. **(B)** Frequency of glioma subtypes according to the 2016 WHO classification in a cohort of 122 patients operated from 2017 to 2021. Glioblastoma IDH-wildtype was the predominant tumor type (n=53), including 3 cases of gliosarcoma, 6 cases of giant cell glioblastoma, and 1 case of epithelioid glioblastoma. Diffuse astrocytoma accounted for 7 cases (5 IDH-mutant and 2 IDH-wildtype); Anaplastic astrocytoma IDH-mutant, represented 5 cases; Oligodendroglioma IDH-mutant with 1p/19q codeletion accounted for 2 cases; 1 case was a Pilocytic astrocytoma and 1 case a Diffuse midline glioma, H3K27-mutant. Indeterminate diagnosis (labeled as NOS) accounted for 51 cases: 40 Glioblastomas, 5 Diffuse astrocytomas, 4 Anaplastic astrocytomas and 2 Anaplastic oligodendrogliomas. **(C)** Frequency of glioma subtypes according to the WHO 2021 classification in a cohort of 99 patients operated from 2022 to 2024. Glioblastoma IDH-wildtype was the most frequent type (n=66). This group included classic glioblastoma IDH-wildtype (n=55) as well as gliosarcoma (n=5), giant cell glioblastoma (n=3), and epithelioid glioblastoma (n=3). Other tumor types included astrocytoma IDH-mutant (n=7), oligodendroglioma IDH-mutant with 1p/19q codeletion (n=1), and pleomorphic xanthoastrocytoma (n=1). Indeterminate diagnoses due to insufficient molecular work-up were categorized in two groups: High-grade (grade 3 and 4) glioma NOS, which accounted for 15 cases, and Low-grade (grade 1 and 2) glioma NOS, which accounted for 9 cases.

According to the 2007 WHO histological classification, in the cohort of patients operated between 2010 and 2016 (n=222), the most frequent subtype was glioblastoma, with 150 cases (68%). This was followed in frequency by oligodendrogliomas (16 cases, 7.2%), anaplastic astrocytomas (12 cases, 5.4%), anaplastic oligoastrocytomas (12 cases, 5.4%), oligoastrocytomas (10 cases, 4.5%) and diffuse astrocytomas (10 cases, 4.5%) and anaplastic oligodendroglioma (8 cases, 3.6%) (**Figure 1**).

**Figure 2:**
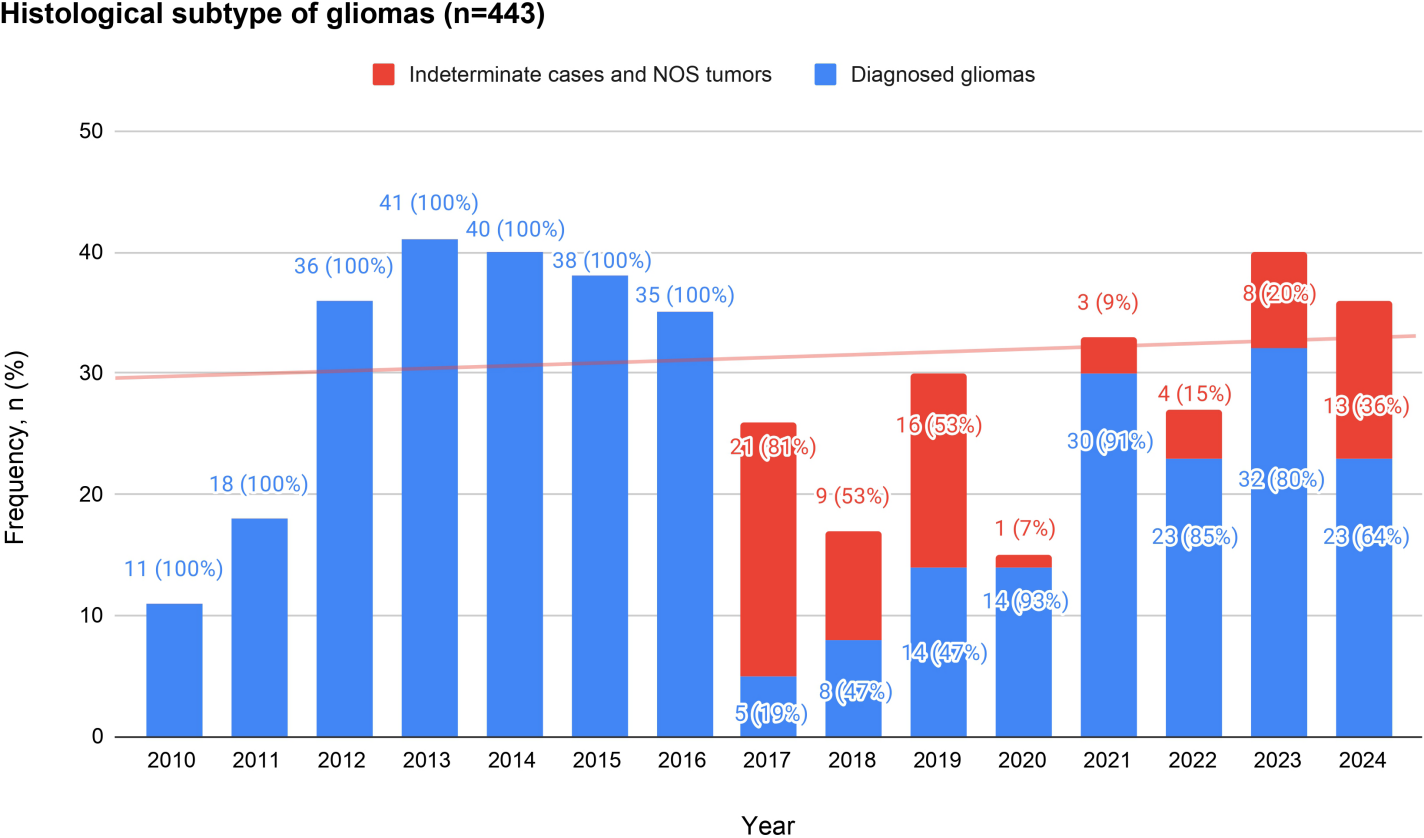
The figure depicts the annual distribution of gliomas operated in our center. Blue bars depict tumors with a definitive diagnosis according to the corresponding WHO’s Classification scheme. Indeterminate diagnoses, classified as “NOS Gliomas”, are depicted in red. Between 2010 and 2016, all cases were histologically categorized, with no indeterminate diagnoses reported. From 2017 onward, coinciding with the introduction of molecular criteria in the WHO classification, there was a progressive increase in NOS cases. NOS cases corresponded to 81% (n=21) in 2017, 53% (n=9) in 2018, 53% (n=16) in 2019, 7% (n=1) in 2020, 9% (n=3) in 2021, 15% (n=4) in 2022, 20% (n=8) in 2023 and 36% (n=13) in 2024.

Between 2017 and 2021 (n=122), with the 2016 WHO classification, which incorporated molecular parameters, the most frequent subtype continued to be glioblastoma IDH-wildtype, with 53 cases (43.0%). During this period, a significant proportion of cases were found to have an incomplete molecular characterization, which caused a peak in NOS tumors. The second most common tumor type was glioblastoma NOS (40 cases, 33.0%). Then followed diffuse astrocytomas, which accounted for 12 cases in total (10%): IDH-mutant (5 cases; 4%), IDH-wildtype (2 cases; 2%), and NOS (5 cases, 4%). Anaplastic astrocytomas represented 9 cases (9%): IDH-mutant (5 cases; 4%) and NOS (4 cases; 3%). Anaplastic Oligodendroglioma IDH-mutant were 2 cases (2%), and Anaplastic Oligodendroglioma NOS were other 2 cases (2%). Pilocytic Astrocytoma and Diffuse Midline Glioma, H3 K27M-mutant were 1 case (1%) each (**Figure 2**).

**Figure 3:**
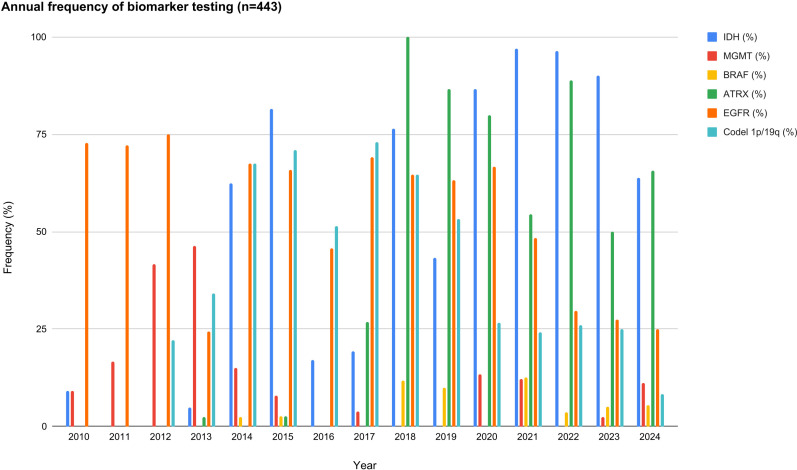
The figure displays the annual frequency of testing (tests performed with either positive or negative results) for the main glioma biomarkers available throughout the study period (2010–2024). It includes IDH1 R132H, *MGMT*, BRAF, *ATRX*, *EGFR*, and 1p/19q codeletion, showing variations in their implementation across different years. There is an evident increase in overall biomarker testing from 2016 onward, but systematic implementation is not reached.

The application of the 2021 WHO classification from 2022 to 2024 (n=99), showed that glioblastoma IDH-wildtype remained the predominant subtype, with 66 cases (67%). This was followed by high-grade glioma-HGG (15 cases, 15%) and low-grade glioma-LGG (9 cases, 9%) tumors. Together (tumors labeled as either HGG or LGG), they represent 24%, and correspond to tumors that could not be categorized within any tumor type due to a lack of molecular data. Astrocytomas, IDH-mutant followed in frequency with 7 cases, 7%. Astrocytoma, IDH-mutant and Pleomorphic xanthoastrocytoma was 1 case; 1% each one (**Figure 3**).

During the period corresponding to the WHO 2007 classification, molecular testing availability was limited and heterogeneous. Between 2010 and 2013, testing was largely restricted to selected immunohistochemical markers, with progressive incorporation of IDH1 R132H immunohistochemistry from 2014 onward. Assessment of *EGFR* amplification was analyzed in 70–75% of cases between 2010 and 2012, but then gradually declined. 1p/19q codeletion testing was first reported in 2012 (22.2%), peaked in 2015 (71.1%), and subsequently decreased. *MGMT* activity displayed a variable pattern, with 9.1% in 2010, rising to 41.7% in 2012 and 46.3% in 2013, and subsequently fluctuating with low values. Regardless of molecular studies, all tumors in this period were classified according to histopathological criteria, and no indeterminate diagnosis were recorded (**Figure 3**).

Following the implementation of the WHO 2016 classification, molecular testing became more frequent but remained variable. IDH testing increased substantially and was performed in the majority of cases by the end of this period, it began at 17% in 2016 and increased to 19% in 2017, followed by an exponential growth in 2018, reaching 76%, and ultimately achieving 87% by 2020. ATRX immunohistochemistry was first tested in 2017, when it was performed at a rate of 27%, showed a peak of 100% in 2018, and remained above 80% in 2019 and 2020 (87% and 80%, respectively). Regarding 1p/19q codeletion, a decrease was observed in 2016 compared with 2015 (51%), followed by a peak in 2017 with a completion rate of 73%, after which a linear decline was noted over the subsequent years (2018: 65%, 2019: 53%, and 2020: 27%). *EGFR* amplification showed a testing rate of 46% in 2016 and then remained relatively stable in the subsequent years (2017: 69%, 2018: 65%, 2019: 63%, and 2020: 67%). Finally, *MGMT* activity testing was performed infrequently, with rates of 4% in 2017 and 13% in 2020 (in 2016, 2018 and 2019 was 0%). During this period, incomplete availability of mandatory molecular markers resulted in the emergence of indeterminate diagnosis due to incomplete molecular profiling (**Figure 3**).

With adoption of the WHO 2021 classification, the requirement for integrated molecular diagnosis increased further. IDH testing remained high in most patients, exceeding 90% from 2021 to 2023 (97%, 96%, and 90%, respectively), before declining to 64% in 2024. ATRX testing showed alternating periods of decrease and increase in testing rates (55% in 2021, representing a decline compared with 2020; 89% in 2022; 50% in 2023; and 66% in 2024).Assessment of 1p/19q codeletion remained relatively stable at approximately 25% from 2021 to 2023 (24%, 26%, and 25%, respectively), followed by a marked decline in 2024 to 8%. *EGFR* amplification showed a decrease compared with the previous classification, with a testing rate of 48% in 2021, and then remained stable from 2022 to 2024 (30%, 27%, and 25%, respectively). *MGMT* activity testing remained limited and inconsistent, with rates of 12% in 2021, no testing performed in 2022, 2.5% in 2023, and 11% in 2024. As a result, a substantial proportion of tumors could not be fully classified according to WHO 2021 criteria and were categorized either as low-grade or high-grade glioma (**Figure 3**).

## Discussion

Over the past decade, molecular profiling has significantly reshaped the characterization of brain tumors and has been progressively incorporated into tumor classification systems. However, the implementation of these molecular techniques remains uneven, and their integration into routine clinical practice has not been equitable across healthcare settings (9). We hypothesized that the progressive incorporation of mandatory molecular markers in successive glioma CNS WHO classifications would be associated with an increased frequency of incomplete tumor characterization due to inadequate access to molecular testing. To address this situation, we analyzed the cases of a high-volume neurosurgical tertiary center in Latin America with specialized neuropathology expertise, advanced diagnostic infrastructure, and sustained exposure to complex neuro-oncological cases.

The transition across WHO editions revealed a progressive change in the proportion of precise diagnoses in this cohort. Between 2010 and 2016, all tumors were histologically classified, with no indeterminate diagnoses recorded. However, starting in 2017—aligned with the implementation of the 2016 WHO classification that incorporated molecular criteria—a steady increase in indeterminate diagnosis and NOS cases was observed, reaching over 50% of diagnoses in some periods (2017: 81%, 2018: 53%, 2019: 53%). In recent years, with the 2021 WHO update, the proportion of tumors with incomplete molecular characterization has remained high, representing 15% in 2022, 20% in 2023, and 36% in 2024, highlighting the difficulty of fully applying the most recent classification (**Figure 3**). This temporal pattern illustrates the transition from a purely histological framework to a more molecular approach, at the expense of a higher proportion of indeterminate diagnoses.

Despite the well-established diagnostic and prognostic importance of IDH, the frequency of its determination in our cohort was irregular throughout the study period. Although an initial increase was observed in 2014–2015, this trend was not consolidated in the following years. In the most recent period, between 2022 and 2024, about one-third of patients still lacked IDH determination, highlighting that this test was not readily available for implementation. ATRX testing showed a more consistent growth from 2017 onward, with frequencies surpassing 70% in some years. In contrast, 1p/19q codeletion exhibited a more inconsistent pattern, with peaks close to 70% in 2015 but significant declines in subsequent years, stabilizing at around 20–30% in the most recent period, reflecting technical and accessibility challenges for its systematic use. *MGMT* testing displayed a heterogeneous trend, with elevated frequencies in the early years (2010–2013) and again between 2017 and 2020, but without becoming a consistent routine practice. Collectively, these limitations resulted in a considerable proportion of indeterminate and NOS diagnoses (10), ultimately restricting both prognostic accuracy and the ability to offer molecularly driven clinical trials and stratification.

Although several key molecular markers can be assessed using relatively straightforward immunohistochemical techniques, their availability in routine clinical practice remains inconsistent and fluctuated over time in our cohort. The observed variations in testing rates, including the decline in IDH assessment in the most recent period, were primarily driven by intermittent shortages of essential reagents and commercial kits required to perform these assays. As a consequence, even routinely implemented immunohistochemical studies could not be performed systematically during certain intervals. Similar structural constraints also affected the implementation of 1p/19q codeletion testing. Its availability was subject to many of the same logistical and economic limitations described for immunohistochemistry, including intermittent shortages of reagents and commercial kits and reimbursement-related restrictions, contributing to the temporal variability observed in testing rates. Nonetheless, an inverse temporal relationship emerged between ATRX immunohistochemical assessment and 1p/19q codeletion testing. As ATRX implementation increased, the frequency of 1p/19q testing declined. This pattern likely reflects a rational diagnostic adaptation, as *ATRX* loss is largely mutually exclusive with 1p/19q codeletion and strongly associated with astrocytic lineage. Given that ATRX immunohistochemistry is less resource-intensive than FISH-based assays, its expanded use may have enabled a more resource-adapted approach to codeletion testing.

On the other hand, comprehensive molecular characterization through sequencing-based or non-immunohistochemical techniques is frequently not reimbursed by health insurance providers in our setting, which further limits their routine use and negatively impacts the completeness of tumor profiling. These economic constraints particularly affect the implementation of advanced molecular assays despite their recognized diagnostic relevance. Furthermore, local capacity for DNA-based methylation analyses is not available, precluding the assessment of *MGMT* promoter methylation throughout the study period, which was in all cases performed by assessing enzymatic activity through IHC. These limitations are further compounded by the absence of standardized methodologies and universally accepted cut-off values for several key biomarkers (10, 11). Although the WHO 2021 classification incorporates essential molecular signatures such as *IDH 1/2*, 1p/19q, *TERT*, and *EGFR*, it does not mandate a minimum diagnostic panel or standardized assays, leading to heterogeneous practices across institutions. *MGMT* promoter methylation exemplifies these challenges (12, 13), as variability in testing methods and thresholds may result in inconsistent interpretation and potential misclassification (10–15).

Consequently, despite the clear increase in molecular testing after the WHO 2016 and especially the 2021 updates, implementation has hardly been universal in our setting. Many patients remained without full profiling (according to their corresponding tumor type), leading to incomplete characterization of their neoplasms. This situation has had a paradoxical effect: while in high-income countries the new WHO classifications may have refined diagnoses, in resource-limited contexts like this they may have as well led to an increase in cases labeled as NOS-Tumors, High or Low grade glioma, because fulfilling the mandatory molecular criteria for a specific tumor type is often unfeasible (12, 16).

These findings are consistent with international reports describing marked disparities in access to molecular diagnostics for gliomas in low- and middle-income countries (LMICs) (6–18). While advanced molecular testing is routinely available in high-income settings, its implementation in resource-limited regions remains restricted by infrastructural constraints, high costs, and limited expertise (16, 17). In Latin America, these challenges are further compounded by socioeconomic inequalities and unequal access to specialized care, particularly outside major urban centers (12–20). Recent regional and international surveys have shown that only a minority of centers in LMICs have access to even basic molecular diagnostics (21), underscoring the gap between current WHO recommendations and real-world diagnostic capabilities.

The lack of molecular diagnosis limits both diagnostic/prognostic accuracy and therapeutic options (22). In these situations, treatment typically consists of the standard combination of surgical resection, radiotherapy, and chemotherapy (like temozolomide), without the possibility of incorporating targeted therapies for molecular subtypes (e.g. IDH inhibitors, BRAF inhibitors), specially at recurrence (23–26). Equally important, a precise molecular profile is now a critical prerequisite for enrollment in many clinical trials. In Latin-America, clinical trials in neuro-oncology are already scarce, and the number of patients actively recruited remains very low (27–29). When molecular testing is incomplete or unavailable, potentially eligible patients may be excluded simply because their tumors cannot be fully characterized according to the inclusion criteria. This further reduces opportunities for access to novel therapies and contributes to the widening gap between patients in resource-limited settings and those in high-income countries, where clinical trial participation is increasingly driven by molecularly defined subgroups (14–32).

A key strength of this study lies in its analysis of a large patient cohort from a leading neurosurgical center in Latin America, which allows for the exploration of a real and pressing issue in daily clinical practice. The study spans multiple versions of the WHO classification, providing an evolutionary perspective on how increasing molecular requirements have impacted diagnostic capacity and the use of data derived from routine clinical care ensures the practical relevance of the findings. Also, all determinations were consistently performed by the same pathologist using automated systems, ensuring a high level of uniformity in the evaluation process. This methodological approach minimizes inter-observer variability and reduces the potential for omissions or misinterpretations. Furthermore, the challenges faced by a high-volume tertiary center such as the one in this cohort serve to some extent as a surrogate for the more marked limitations that may arise in smaller centers or those with more limited resources in the region.

The fifth edition of the WHO classification of CNS tumors (CNS5) has reinforced the need for routine integration of molecular biomarkers in the diagnosis of gliomas, not only to reduce histopathological ambiguity but also to improve prognostic stratification and guide more personalized treatment strategies (4–33). Nevertheless, the accelerated incorporation of advanced diagnostic tools such as molecular profiling sets global standards that are difficult to meet in resource-constrained settings and even in high-volume neuro-oncology centers like ours. This raises a fundamental dilemma: should countries with limited access continue pursuing a care model based on still-inaccessible technologies, or should research be reoriented toward alternative, context-adapted, and sustainable solutions? Raising this question does not imply rejecting scientific progress, but rather advocating for strategies that allow high-quality diagnosis and treatment using the resources currently available. Studies like this one can provide evidence to inform public policies, reprioritize institutional strategies, and promote research agendas that are locally relevant without falling behind in the global scientific paradigm.

## Limitations

The retrospective design of this study introduces inherent limitations, such as variability in the availability of molecular markers over time and the possible omission of data not recorded in clinical charts. Additionally, this is a single-center study, which may limit the generalizability of the findings to other institutions. Lastly, this study does not assess correlations between diagnostic precision and survival or other clinical outcomes—an aspect that could be explored in future research.

## Data Availability

The raw data supporting the conclusions of this article will be made available by the authors, without undue reservation.
