# Correction: Accessibility to molecular studies in gliomas: a challenge for precision medicine in low-resource countries

**DOI:** 10.3389/fonc.2026.1897498

**Published:** 2026-06-09

**Authors:** Pablo S. Paolinelli, Tomas Saavedra Azcona, Florencia BCasto, Ezequiel Jungberg, Nicolás Tilano, William ABlettler, Joan SPazos, Silvina Dell’Era, Clara Lynch, Miguel Villaescusa, Pedro Plou, Pablo Ajler

**Affiliations:** 1 Neurosurgical Department, Hospital Italiano de Buenos Aires, CABA, Buenos Aires, Argentina; 2 Research Department, Instituto Universitario Hospital Italiano de Buenos Aires, CABA, Buenos Aires, Argentina; 3 Faculty of Engineering, Universidad Austral, Pilar, Buenos Aires, Argentina

**Keywords:** glioma, Latin America, low resources, molecular diagnostics, molecular parameters, precision medicine, WHO classification

An incorrect **Funding** statement was provided. The correct **Funding** statement reads:

“The author(s) declared that financial support was received for this work and/or its publication. Servier Argentina provided financial support to cover the article processing charge (APC). The funder was not involved in the study design, collection, analysis, interpretation of data, the writing of this article, or the decision to submit it for publication”.

The original version of this article has been updated.

